# Nivolumab as adjuvant treatment for a spinal melanocytoma: A case report

**DOI:** 10.1097/MD.0000000000025862

**Published:** 2021-05-14

**Authors:** Virginie Hean, Wafa Bouleftour, Carole Ramirez, Fabien Forest, Claire Boutet, Romain Rivoirard

**Affiliations:** a Service de Neurologie, CHU de Saint-Etienne, Hôpital Nord, Avenue Albert Raimond, Saint Etienne CEDEX 2; b Département d’Oncologie Médicale, Institut de Cancérologie Lucien Neuwirth, Saint-Priest-en-Jarez; c Laboratoire d’Anatomie et Cytologie Pathologiques, CHU de Saint-Etienne, Hôpital Nord, Avenue Albert Raimond, Saint Etienne CEDEX 2; d Service de Radiologie, CHU de Saint-Etienne, Hôpital Nord, Avenue Albert Raimond, Saint Etienne CEDEX, France.

**Keywords:** GNAQ mutation, immunotherapy, melanocytoma

## Abstract

**Rationale:**

Meningeal melanocytoma is a rare benign melanocytic tumor of the central nervous system. We report for the first time a case of meningeal melanocytoma treated with immunotherapy.

**Patient concerns:**

A 70-year-old man with no medical history was admitted to the Emergency Room. He suffered from a motor and sensory deficit in his left lower limb and a bilateral upper arm neuralgia.

**Diagnoses:**

A contrast-enhanced magnetic resonance imaging (MRI) was performed. It showed a C7-T1 bleeding intramedullary tumor. Laminectomy was decided and performed. The results of the pathologic examination showed a melanocytic tumor harboring GNAQ mutation. Meningeal melanocytoma was the final diagnosis.

**Interventions:**

The patient was treated with 10 radiotherapy sessions and 6 cycles of nivolumab. A year later, the patient experienced neuralgia again with severe pain and an increasing sensory motor deficit. He underwent a second surgery that was incomplete. As the tumor kept growing, he received temozolomide. But the 6th cycle had to be interrupted due to bedsore infection in the hip area.

**Outcomes:**

Disease progression finally led to the patient's death 3 years after diagnosis.

**Lessons:**

This case report is the first about a patient with meningeal melanocytoma treated with immunotherapy. Treatment based on biomolecular mutations will probably change spinal melanocytoma therapeutic approach in the next few years.

## 1 Introduction

Meningeal melanocytoma is a rare benign melanocytic tumor of the central nervous system (CNS).^[1]^ It is derived from leptomeningeal melanocytes and mostly occur along the posterior fossa. Magnetic Resonance Imaging (MRI) is quite typical with a paramagnetic behavior due to the melanin in the cells. Melanic tumors of the CNS are rare and are often mistaken with melanoma metastases.^[2]^ Limas and Tio first reported a primary melanotic benign tumor of the meninges in 1972.^[3]^ Due to the limited number of cases, it is difficult to calculate the incidence of such tumors.^[4]^ They are mainly located at the sulci around the base of the brain and the upper cervical spinal cord^[5]^ and they are highly recurrent.^[6]^ So far, no guideline has been provided to treat such tumors.

## 2 Case report

**Figure 1:**
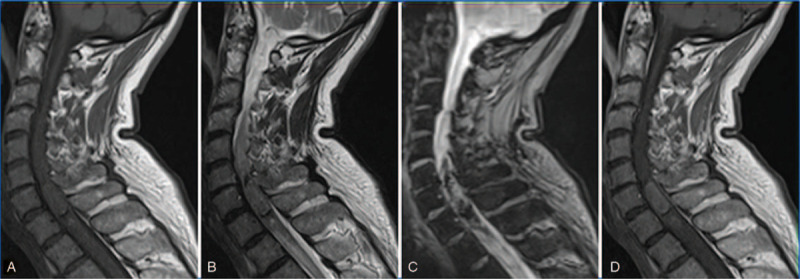
Cervical medullar magnetic resonance imaging, sagittal plane. The intra medullar lesion is hyperintense on T1-weighted sequence (A), isointense on T2-weighted sequence (B), presents signs of bleeding on T2^∗^-weighted sequence (hypointensity) (C) and homogeneous enhanced with gadolinium injection (D).

We report the case of a 70-year-old man with no medical history. After experiencing a motor sensory deficit in his left lower limb for 24 hours, he went to the emergency room (ER). He also reported he had been suffering from bilateral upper arm neuralgia for over a year. A contrast-enhanced medullary MRI showed a bleeding intra medullary tumor next to C7-T1 (Fig. 1). A left cerebellar tonsil lesion was also viewed.

The initial work-up was completed by a chest, abdomen and pelvis (computerized tomography) scan as well as and a PET-TDM (Positron Emission Tomography): no sign of metastatic spread was evidenced. The C7-D2 intramedullary lesion from hypermetabolic (SUV 5.2). A skin melanoma was ruled out after a dermatology consultation as no cancer lesion was seen during skin and mucosal examination.

**Figure 2:**
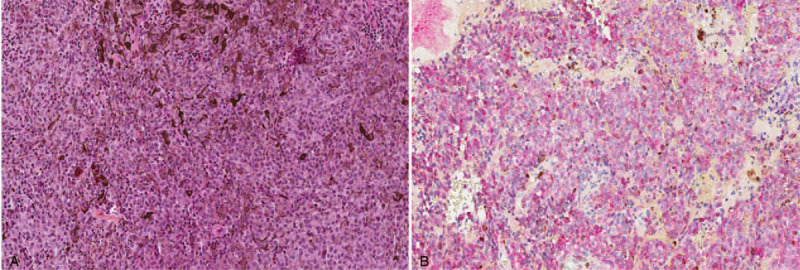
Hematoxylin & Eosin, x200: pleormophic tumor cells with melanin. Immunohistochemistry anti-Melan-A, x200: diffuse staining of tumor cells.

First, the tumor was removed on 7/12/2016 but surgery did not solve the motor deficit problem. The patient left the hospital and had home physical therapy sessions. The pathology report leaned towards a melanocytic tumor without mitotic figures and with low Ki67proliferation index (Fig. 2). The molecular study showed p.Q2019L mutation of GNAQ, an elective mutation in uveal and meningeal melanocytic tumors.Secondly, the patient received 30 Gy in ten sessions of radiotherapy, from 8/19/2016 to 9/1/2016. He also had 6 cycles of nivolumab (3 mg/kg), from 9/7/16 to 11/23/16, representing a total dose of 1260 mg. A disease stability of 13 months was observed following immunotherapy. The patient developed a postimmunotherapy hypothyroidism that was treated with levothyroxine.

During follow-up, MRIs revealed a persistent growing residual tumor. One year after the last nivolumab injection, the patient experienced a very painful brachial neuralgia which led him to the ER again. The MRI performed there showed the tumor had progressed. Thus, the patient was hospitalized and underwent a second surgery on 12/27/17. The postop MRI showed no residual tumor and no rachis hematoma. Pathological examination revealed a meningeal melanocytoma similar to the first 1, with Ki <5%.Then, the patient spent a few months in a rehabilitation center.

Three months later, the MRI evidenced a tumor recurrence. In the following months, the tumor kept growing. In December 2018, a national multidisciplinary team meeting dedicated to spinal cord tumors decided on 3 cycles of chemotherapy by temozolomide. Treatment was started in April 2019 as the MRI showed the tumor had stopped growing. Three additional cycles of temozolomide were prescribed. The patient received a total dose of 7720 mg of temolozomide. Chemotherapy first cycles were initiated at a dose between 150 and 200 mg/m^2^. This dosage was reduced to 100 mg/m^2^ the last 2 cycle due to toxicity associated with an alteration of patient general condition. Despite local symptomatic treatment, the patient suffered from bedsores in the sacrum area so he went to the ER in a local hospital. After a few days, the bedsores became infected so the patient was admitted in the dermatology unit in a teaching hospital. There, he was given piperacillin and amikacin. The sixth cycle of temozolomide was then interrupted on 9/12/2019.

The patient's condition got worse: his need of oxygen increased and a lower limbs oedema occurred. A thoracic computerized tomography angiography showed a right proximal pulmonary embolism for which he spent a short time in the Intensive Care Unit (ICU) and had surgical debridement. The patient died a few days after returning in the dermatology unit.

## 3 Discussion and conclusions

Meningeal melanocytoma is a rare brain tumor,^[7]^ it is part of benign leptomeningeal tumors. It is mainly located in the posterior fossa, but in the case in this clinical report the tumor was located at spinal level.^[8]^ To diagnose such tumors, pathology examination that is to say microscopical examination, immunochemistry analysis and molecular study are gold standard tests.^[9]^ This tumor mostly affects women, it is often diagnosed at the age of 50 years old^[10]^ yet, rare cases affect kids.^[11]^ Due to a limited number of cases, it is difficult to calculate the incidence of such tumors.^[4]^

MRI images usually show an increase of T1 signal intensity as well as T2 isointensity or hypointensity.^[2]^ The intensity of the signal depends on the tumor concentration in melanin. Contrast enhancement is homogeneous. This tumor is mainly located in the brain and the upper cervical spinal cord even though it can be anywhere within the CNS. It can be mistaken with a melanotic meningioma. The symptoms that lead patients to consult a doctor can be extremely different, depending on the tumor location.^[4]^ In their study, Keng-Liang et al reported 12 patients with meningeal melanocytoma and Nevus of Ota^[12]^ thus every patient should have a complete dermatology examination. In the case of our patient, he was Nevus of Ota-free.

Rades et al conducted a retrospective study about 89 patients with meningeal melanocytoma in order to compare patients’ different treatments.^[6]^ They found that complete resection followed by radiotherapy seemed to improve the outcome unlike incomplete resection or no radiotherapy. In another study, Rades et al concluded that complete tumor resection with postsurgery radiotherapy should be considered the best therapeutic option.^[13]^

Since the incidence cannot be calculated, it is impossible to conduct prospective studies. Moreover, treatments can only be analyzed retrospectively. Identifying biomolecular mutations can help to choose the most adequate treatment since multidisciplinary team meetings now tend to take into account the tumor genomic rather than its location. The mutations in meningeal melanocytoma are different from those in melanic tumors (RAS/RAF path), they are G protein mutations.^[14,15]^

Programmed cell death 1 (PD-1) is expressed on the surface of immune cells in the cerebrospinal fluid. Recently, Portnow et al study demonstrated that intravenous pembrolizumab (PD-1 blocking antibody) were sufficient for blocking PD-1 on endogenous and adoptively transferred T cells in the brain.^[16]^ To date, only 1 phase I study still in the process of recruiting patients, tests immunotherapy in patients with leptomeningeal metastases of melanoma including melanocytoma. The preliminary results of this study which included 2 patients with primary central nervous system tumor demonstrated that concurrent intrathecal and intravenous nivolumab administration is safe with no significant toxicities.^[17]^

This case report is the first about a patient with meningeal melanocytoma treated with immunotherapy. Such tumors may evolve from a long time malignant transformation to a rapid massive meningeal diffusion.^[18]^ On the whole, these tumors are considered a good prognosis even though they can be locally aggressive or transform into malignant tumors.^[19]^ Therefore, it is crucial to reach a rapid diagnosis. When a clinician comes across a spinal melanotic tumor, he should think about primitive tumors as metastases are not the only possible diagnosis.

We report a case of a meningeal melanocytoma treated by surgery, radiotherapy and immunotherapy (checkpoint inhibitor) at initial diagnosis. The patient died because of the complications due to tumor evolution. Although such a tumor is benign, it must be investigated and treated at an early stage to avoid complications. Treatment based on biomolecular mutations will probably change the therapeutic approach in the next few years.

## Acknowledgments

The authors would like to thank Sandrine Sotton for English editing services. Miss Sandrine Sotton gives permission to be named.

## Author contributions

**Conceptualization:** Romain Rivoirard.

**Supervision:** Romain Rivoirard, Carole Ramirez.

**Validation:** Romain Rivoirard.

**Writing – original draft:** Virginie Hean, Wafa Bouleftour.

**Writing – review & editing:** Wafa Bouleftour, Carole Ramirez, Fabien Forest, Claire Boutet, Romain Rivoirard.
